# Non‐Solvent Induced Phase Separation Enables Designer Redox Flow Battery Electrodes

**DOI:** 10.1002/adma.202006716

**Published:** 2021-03-02

**Authors:** Charles Tai‐Chieh Wan, Rémy Richard Jacquemond, Yet‐Ming Chiang, Kitty Nijmeijer, Fikile R. Brushett, Antoni Forner‐Cuenca

**Affiliations:** ^1^ Joint Center for Energy Storage Research Massachusetts Institute of Technology 77 Massachusetts Avenue Cambridge MA 02139 USA; ^2^ Department of Chemical Engineering Massachusetts Institute of Technology 77 Massachusetts Avenue Cambridge MA 02139 USA; ^3^ Membrane Materials and Processes Department of Chemical Engineering and Chemistry Eindhoven University of Technology P.O. Box 513 Eindhoven 5600 MB The Netherlands; ^4^ Dutch Institute for Fundamental Energy Research (DIFFER) P.O. Box 6336 Eindhoven 5600 HH The Netherlands; ^5^ Department of Materials Science and Engineering Massachusetts Institute of Technology 77 Massachusetts Avenue Cambridge MA 02139 USA

**Keywords:** energy storage, phase separation, porous electrodes, redox flow batteries

## Abstract

Porous carbonaceous electrodes are performance‐defining components in redox flow batteries (RFBs), where their properties impact the efficiency, cost, and durability of the system. The overarching challenge is to simultaneously fulfill multiple seemingly contradictory requirements—i.e., high surface area, low pressure drop, and facile mass transport—without sacrificing scalability or manufacturability. Here, non‐solvent induced phase separation (NIPS) is proposed as a versatile method to synthesize tunable porous structures suitable for use as RFB electrodes. The variation of the relative concentration of scaffold‐forming polyacrylonitrile to pore‐forming poly(vinylpyrrolidone) is demonstrated to result in electrodes with distinct microstructure and porosity. Tomographic microscopy, porosimetry, and spectroscopy are used to characterize the 3D structure and surface chemistry. Flow cell studies with two common redox species (i.e., all‐vanadium and Fe^2+/3+^) reveal that the novel electrodes can outperform traditional carbon fiber electrodes. It is posited that the bimodal porous structure, with interconnected large (**>**50 µm) macrovoids in the through‐plane direction and smaller (**<**5 µm) pores throughout, provides a favorable balance between offsetting traits. Although nascent, the NIPS synthesis approach has the potential to serve as a technology platform for the development of porous electrodes specifically designed to enable electrochemical flow technologies.

## Graphical abstract

Non‐solvent induced phase separation is introduced as a versatile and facile method to synthesize redox flow battery electrodes with hierarchical microstructures unachievable via conventional manufacturing approaches. Flow cell studies reveal that the novel materials can outperform conventional carbon fiber electrodes owing to better mass transfer and higher surface area, demonstrating that this method may enable new classes of porous electrodes tailored for particular electrochemical flow technologies.

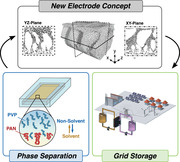

Stationary energy storage is poised to play a pivotal role in the efficient delivery of electricity, particularly for the integration of low‐cost, variable, and sustainable energy sources into the electricity grid.^[^ ^1^, ^2^ ^]^ Redox flow batteries (RFBs) are a promising technological option for multihour energy storage owing to their decoupling of energy and power rating, scalability, and longer operational lifetimes.^[^ ^3^ ^]^ Compared to sealed electrochemical devices, RFB electrolyte tanks are easily accessible, enabling electrolyte scale‐up, maintenance, and potential exchange of new redox couples (**Figure** **1**a). Despite their advantages, current iterations of RFBs are considered too costly for many emerging grid applications,^[^ ^1^, ^4^, ^5^ ^]^ motivating research into improved electrolyte formulations,^[^ ^6^, ^7^ ^]^ separation technologies,^[^ ^8^, ^9^, ^10^ ^]^ operational strategies,^[^ ^11^ ^]^ and materials design.^[^ ^12^ ^]^ In particular, increasing power density enables more compact and efficient reactors that can meet operational demands, reducing electrochemical stack size and costs. Within the reactor, the porous carbonaceous electrode supports several important functions, including conducting electrons and heat, providing surface area for redox reactions to occur, distributing electrolyte through the reactor, and regulating the operational pressure drop.^[^ ^13^ ^]^ Thus, the interfacial and microstructural properties influence electrochemical and fluid dynamic performance, ultimately impacting system efficiency and cost.^[^ ^14^ ^]^ Historically, conventional RFB electrodes have been fibrous mats derived from polyacrylonitrile (PAN) precursor and assembled into coherent structures including papers, cloths, or felts.^[^ ^15^ ^]^ Such formats are functional for convection‐driven electrochemical technologies owing to their permeability (*k* ≈ 10^−10^ to 10^−12^ m^2^), (electro)chemical stability, and electronic conductivity. Each unique fiber arrangement results in constructs with idiosyncratic microstructural features, translating to disparities in available surface area, pore size distribution, fluid dynamics, and consequently, electrochemical performance.^[^ ^16^ ^]^ The most commonly used materials are repurposed from fuel cell gas diffusion electrodes (GDEs) and are not designed to support the liquid‐phase electrochemistry of contemporary RFBs. Advances in zero‐gap reactor architecture and thinner electrodes to ameliorate ohmic losses have led to significant performance enhancement at the lab scale;^[^ ^14^, ^17^ ^]^ furthermore, reactor design and stack topology are continually progressing to satisfy larger‐scale energy demands.^[^ ^18^, ^19^, ^20^, ^21^, ^22^ ^]^ However, the fundamental challenge remains to simultaneously enable ample surface area for redox reactions, high permeability to reduce the pressure loss, and augmented mass transfer to minimize concentration overpotentials—all while using materials that are fabricated using continuous, low‐cost, and sustainable manufacturing techniques.

**Figure 1:**
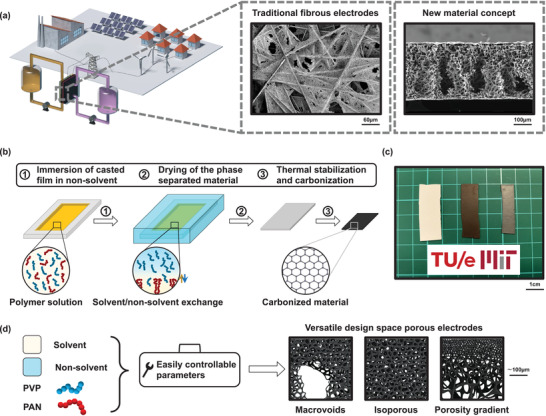
Concept for NIPS‐fabricated porous electrodes integrated into a RFB. a) RFB powered by phase separated electrodes connected to electric grid and intermittent renewable energy sources. Inset view: SEM top view of SGL29AA (left) and SEM cross section of PSP‐2:3 (right). b) Schematic representation of the different steps involved in the production of flat sheet carbonized materials using phase separation. c) Optical photographs of the phase separated materials at different stages of the preparation process. Left: after drying (≈1.1 mm thick), middle: after thermal stabilization (≈1 mm thick), right: after carbonization (≈0.7 mm thick). d) The use of NIPS with easily controllable parameter to create varieties of porous microstructures. Bottom panel right: Illustration of the different classes of microstructures obtained by NIPS.

Accordingly, research efforts have focused on tailoring RFB electrodes, both in terms of surface functional groups and microstructural arrangements. Interfacial engineering efforts have predominantly comprised introducing elementally diverse functional groups for enhanced wettability and electrochemical activity through thermal, chemical, or other modes of oxidation.^[^ ^13^, ^23^, ^24^, ^25^, ^26^, ^27^ ^]^ In regard to microstructure, a growing body of work focuses on developing interconnected pores across multiple length scales to balance kinetics and mass transport,^[^ ^28^ ^]^ either by fabricating thinner electrospun fiber scaffolds^[^ ^29^ ^]^ with directional fiber alignment^[^ ^30^, ^31^ ^]^ or generating architectures with multitudes of pore sizes.^[^ ^32^, ^33^, ^34^, ^35^ ^]^ Advanced numerical simulations on porous media have demonstrated that streamwise‐oriented fibers and pores can lead to increased permeabilities and improved dispersion efficiency;^[^ ^36^ ^]^ furthermore, material sets possessing wide variability in pore sizes with high specific surface area induce higher dispersion and reaction rates, improving overall performance.^[^ ^37^, ^38^, ^39^, ^40^, ^41^ ^]^ Collectively, these prior works constitute important advances in the electrochemical science and engineering of porous electrodes. However, translation beyond the lab‐scale remains a concern, as constraints inherent to existent large‐scale carbon fiber manufacturing processes necessitate the introduction of additional and often complex post‐treatments to produce electrodes with suitable performance characteristics, which results in increased production costs. Roll‐to‐roll carbon fiber based electrode manufacturing includes a combination of consecutive steps including fiber spinning, sizing, chopping, papermaking/weaving, impregnation, and curing, among others.^[^ ^42^ ^]^ From a manufacturing perspective, the ability to generate diverse 3D porous architectures without using complex fabrication steps is appealing for improved cost efficiency and performance.

Here, we introduce non‐solvent induced phase separation (NIPS) as a novel approach to fabricating RFB electrodes. NIPS enables the generation of nonfibrous porous materials with long‐range interconnected microstructures and unique property profiles—achievable through systematic variation of easily adjustable design parameters (Figure 1b,d)—which are unattainable in current fibrous materials. Such synthetic control opens possibilities toward designing complex interconnected porous networks including spatial porosity gradients or multimodal pore size distributions. Accordingly, electrodes with complex pore profiles may be achieved in a single manufacturing NIPS process instead of several distinct fiber production processes. Furthermore, the NIPS synthesis process is scalable, as demonstrated by existing membrane production facilities with similar process steps.^[^ ^43^ ^]^ In addition to membrane separations,^[^ ^44^ ^]^ NIPS has been demonstrated for preparing electrodes in supercapacitors^[^ ^45^, ^46^ ^]^ and in Li‐ion batteries^[^ ^47^ ^]^ or for electrosensing applications.^[^ ^48^ ^]^ However, to the best of our knowledge, prior efforts have not focused on using NIPS to synthesize porous electrodes suitable for electrochemical systems with forced convection. We hypothesize that the hierarchical combination of highly accessible pores along with areas of finer porosity enables low pressure drop through‐plane highways to distribute liquid electrolyte, leading into higher surface area microvoids, beneficial for RFBs, as previously demonstrated.^[^ ^16^, ^28^, ^49^, ^50^ ^]^ In this interconnected porous network, the pores exhibiting the largest features mitigate convective mass transport losses by replenishing the electrolyte to the high‐surface‐area region, corresponding to zones of finer porosity, reducing the diffusion length, and in turn alleviating activation and mass transport overpotential losses.

Here, we show that by varying the ratio of scaffold forming polyacrylonitrile (PAN) to pore‐forming poly(vinylpyrrolidone) (PVP) in the NIPS casting solution, we create a class of materials with related but differing property sets and, consequently, electrochemical performance. A suite of spectroscopic, microscopic, and electroanalytical methods were used to systematically characterize the properties of the newly synthesized materials, and these results were compared to a commercial standard (SGL 29AA, 186 ± 4 µm). Subsequently, the electrochemical and fluid dynamic performance of all the electrodes were evaluated in diagnostic flow cells with well‐understood and commercially relevant redox electrolytes. Our results show that the unoptimized NIPS electrodes outperform the SGL 29AA electrode owing to reduced kinetic and mass transport overpotentials. Even in their preliminary embodiment, these materials show considerable promise for high power operation. We envision that the NIPS approach has the potential to serve as a scalable synthetic platform that enables the generation of diverse structures for deepening understanding of microstructure–function–performance relations and for progressing performance of emergent electrochemical technologies.

The phase separation process (PSP) yields uniform electrodes, which are illustrated in Figure 1c. Full synthesis details and images are provided in Section S2 and Figure S1 in the Supporting Information. Briefly, a viscous mixture of PAN and PVP dissolved in *N*,*N*‐dimethylformamide (DMF) solvent was first cast in a glass mold. The cast mixture was then immersed in a water bath to initiate phase separation into a polymer‐rich and polymer‐lean phase, whereby the water‐soluble PVP leached into the non‐solvent, leaving behind a porous PAN scaffold. Subsequently, the scaffold was thermally stabilized in air for 1 h at 270 °C at a ramp rate of 2 °C min^–1^, and carbonized under flowing nitrogen for 40 min at 850 °C and 40 min at 1050 °C, with a 5 °C min^–1^ ramp rate in between. The selected conditions were based on well‐documented protocols to improve the mechanical and electrical properties of the resulting electrode.^[^ ^29^, ^51^, ^52^, ^53^ ^]^ The yield after carbonization was ≈57 ± 5% (Figure S2, Supporting Information), in accordance with previously reported values.^[^ ^54^, ^55^ ^]^ The process versatility is captured in Figure 1d; we envision that polyporous, isoporous, and/or porosity gradient electrodes can be fabricated through variation of a range of easily accessible parameters including polymer concentration, bath temperature, and solution viscosity.^[^ ^44^, ^46^, ^56^, ^57^, ^58^ ^]^ Such parameters influence the thermodynamics and kinetics of the phase separation process which ultimately governs the final electrode microstructure. In this proof‐of‐concept study, we elected to focus on the effect of relative amounts of PAN to PVP dissolved in a fixed volume of DMF, investigating three different mixture ratios. For brevity, the electrodes derived from these samples are referred to as PSP‐1:1, PSP‐3:4, and PSP‐2:3, where PSP indicates phase‐separated materials, and the ratio is the relative PAN:PVP amount by mass.

Scanning electron microscopy (SEM) was performed to visualize the synthesized microstructures (**Figure** **2**a). The images reveal highly porous structures, containing regions of large, finger‐like (>100 µm) voids (hereon referred to as macrovoids) interconnected to and outlined from porous networks consisting of smaller cavities (hereon referred to as microvoids). The hierarchical structure is a direct consequence of deliberately choosing polymer concentration ranges that form macrovoid regions during phase separation, which we hypothesized would act as channels to distribute electrolyte and mitigate transport losses.^[^ ^16^, ^59^ ^]^ X‐ray tomography (XTM) was performed to assess the 3D structure of the porous material. An example 3D reconstruction for the PSP‐2:3 is illustrated in Figure 2b; reconstructions of all the samples are shown in Figure S5 (Supporting Information). Although the representative cross section in the *XY*‐plane (Figure 2b) shows the same through‐plane view as the SEM in Figure 1a, the *XZ*‐plane reveals the presence of an internal honeycomb‐like distribution of macrovoids throughout the porous structure, observable in all the PSP materials (Figure S5, Supporting Information).

**Figure 2:**
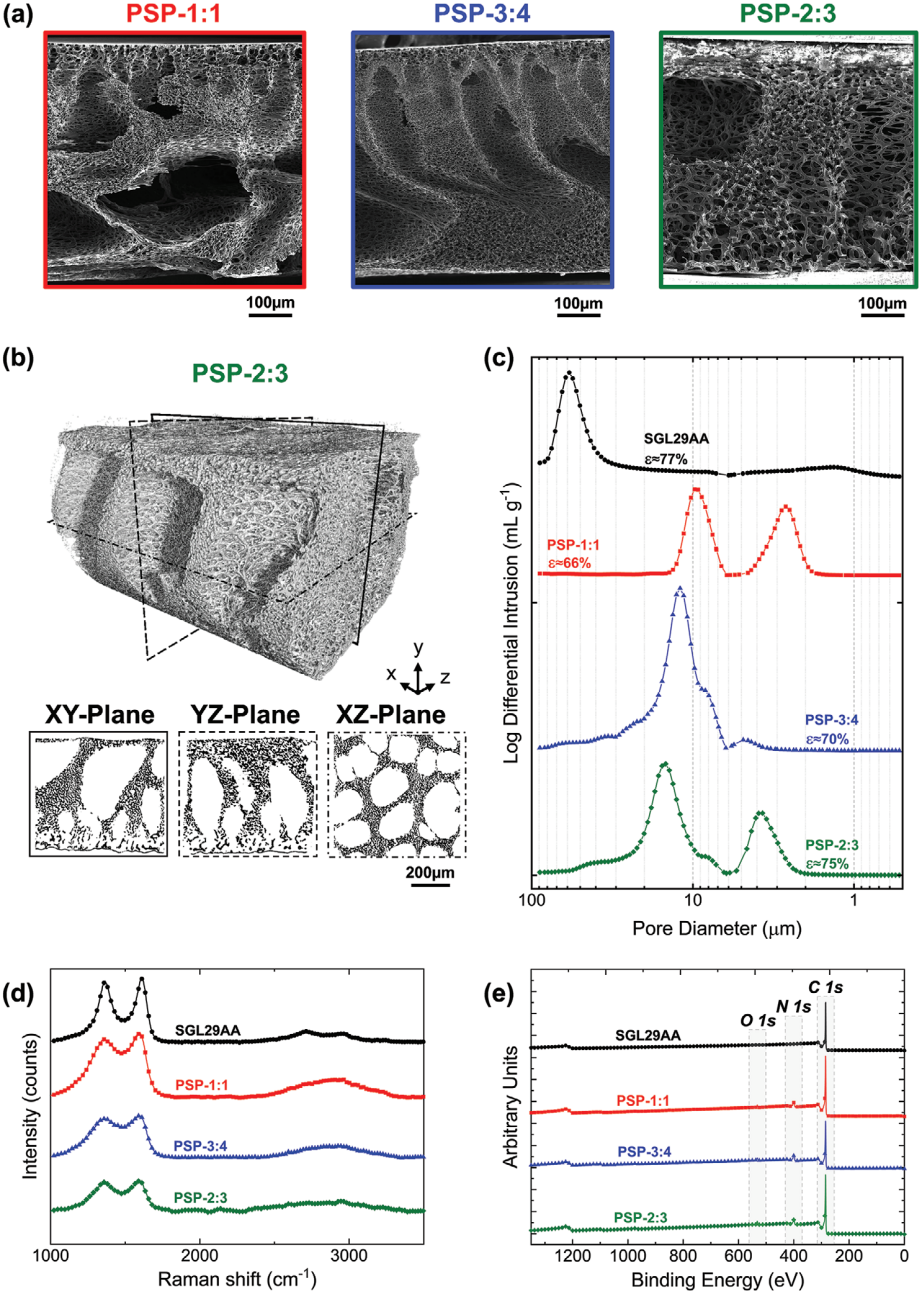
Microstructural and surface chemistry characterization. a) SEM images of PSP‐1:1 (red), PSP‐3:4 (blue), and PSP‐2:3 (green). b) 3D reconstruction of PSP‐2:3 captured by X‐ray tomography (top) with cross‐sectional views in the *XZ*, *YZ*, and *XY*‐planes (bottom). XTMs of all three PSP materials can be found in Figure S5 (Supporting Information). c) Pore size distribution extracted from mercury intrusion porosimetry. Only the microvoids regions (pore diameter < 100 µm) could be resolved, as the macrovoids of the PSP electrodes were not quantifiable owing to the limitations of MIP. d) Raman spectroscopy. e) X‐ray photoelectron spectroscopy. Physical parameters extracted from the microstructural and surface chemistry analysis can be found in Tables S1 and S2 (Supporting Information).

Mercury intrusion porosimetry (MIP) was used as a semiquantitative technique to extract the pore size distributions (PSDs) and porosities (Figure 2c) of each sample in the microporous region. Owing to intrinsic limitations in the observable pore sizes from MIP, macrovoids were not detectable.^[^ ^60^ ^]^ The MIP determined porosities (ε_MIP_) of the PSP materials increased as the relative PVP content increased, whereby PSP‐1:1 had the lowest porosity (ε_MIP_ ≈ 0.66), followed by the PSP‐3:4 (ε_MIP_ ≈ 0.70), and the PSP‐2:3 (ε_MIP_ ≈ 0.75), approaching that of the representative fibrous material SGL 29AA (ε_MIP_ ≈ 0.77). The microvoids of the PSP electrodes ranged from 1 to 60 µm (Table S1, Supporting Information). In all PSP materials, the pore diameters were significantly smaller than that of the SGL 29AA electrode. As expected, PSP‐1:1 with the highest PAN content resulted in narrower PSD (≈1–8 µm) compared to PSP‐2:3 (≈2–50 µm). The PSP‐1:1 and PSP‐2:3 samples exhibited bimodal porous structures; interestingly, the PSP‐3:4 had a predominantly unimodal PSD with a shoulder, resembling the larger pore diameter peak in the PSP‐2:3 sample, but without the additional, distinct smaller pore diameter peak. The unimodal PSD observed from MIP for the PSP‐3:4 was reproducible for multiple samples (Figure S6, Supporting Information). To validate the MIP results, PSDs were also extracted from the SEM images using ImageJ software (Figure S3, Supporting Information). The full extraction procedure is described in Section S2 (Supporting Information). PSDs obtained from SEM images were in good agreement with MIP, although we were unable to resolve the bimodal nature of the microvoids, presumably due to loss of information during the extraction procedure.^[^ ^61^ ^]^ Some samples showed variations in pore size across the electrode thickness (Figure S4, Supporting Information), with smaller pores (corresponding to higher surface area) closer to the membrane, which could be beneficial for balancing transport phenomena with higher electrochemical activity near the membrane.

Raman spectroscopy (Figure 2d) and X‐ray photoelectron spectroscopy (XPS) analysis (Figure 2e) were performed to assess the surface chemistry of the synthesized materials. Raman spectra of SGL 29AA and PSP electrodes exhibited typical fingerprints of carbonaceous materials.^[^ ^62^, ^63^, ^64^ ^]^ The first‐order region spreads from 1100 to 1800 cm^–1^ and the second‐order region from 2200 to 3400 cm^–1^.^[^ ^62^ ^]^ All‐four samples displayed D and G bands around 1380 and 1600 cm^–1^ in the first‐order region and broad bands in the second‐order region attributed to S1 and S2.^[^ ^62^, ^63^ ^]^ The *I*_D_/*I*_G_ ratio was calculated from the Raman spectra, and the values are indicated in Table S2 (Supporting Information). No significant differences were observed which might underline similar “defect” loading in each sample. The presence of binder material in the commercial SGL 29AA might also alter the defect loading, thus stymying a strict comparison of the *I*_D_/*I*_G_ ratios owing to different synthetic conditions. The carbonization conditions along with heteroatoms doping greatly influence the *I*_D_/*I*_G_ ratio; therefore, a deconvolution of the defects generated by the presence of different carbon allotropes or owing to heteroatoms doping remains challenging. When looking at the second‐order region, the PSP electrodes exhibited a broad merged S1/S2 band, whereas SGL 29AA had two distinguishable bands. Previous work suggests that carbon materials exhibiting sharp S1/S2 bands reflect a higher tridimensional order of the graphitic structure which is correlated with high carbonization temperatures, typically close to or higher than 1500 °C.^[^ ^62^, ^63^ ^]^ The SGL 29AA carbon paper shows a low heteroatom content and highly graphitic carbon signature suggesting that high carbonization temperatures (>2000 °C) are used during the carbon fiber manufacturing process, resulting in conductive graphitized materials.^[^ ^55^ ^]^ In comparison, the PSP electrodes exhibit higher heteroatoms content (N at% from 7.8 ± 1.0 to 11.9 ± 2.8 and O at% from 2.9 ± 1.0 to 3.1 ± 1.1) (Table S2, Supporting Information) owing, at least in part, to the lower carbonization temperature (1050 °C) and in accordance with prior reports.^[^ ^52^, ^65^, ^66^ ^]^ Detailed XPS fitting of C1s, N1s, and O1s indicates the PSP electrodes exhibited several nitrogen and oxygen types (Figure S7, Supporting Information). The main nitrogen type groups are ascribed to pyrrolic and pyridinic nitrogen, whereas oxygens are attributed to carbonyl and ether/epoxy/alcohol groups. The presence of quaternary, pyrrolic, and pyridinic nitrogen types is corroborated with the cyclization of PAN. We emphasize that using a lower carbonization temperature leads to a greater density of desirable electrocatalytic heteroatoms, comparable effective conductivity, and potentially reduced production costs by eliminating the need to use expensive high temperature ovens.^[^ ^54^ ^]^

To demonstrate the performance of the synthesized materials under conditions closer to practical embodiments, we first use a single‐electrolyte flow cell configuration (**Figure** **3**a) to assess cell polarization and to quantify resistive losses using electrochemical impedance spectroscopy (EIS) at steady‐state conditions and 50% state‐of‐charge (SoC).^[^ ^14^, ^16^, ^67^ ^]^ The single‐electrolyte setup is advantageous because it enables the identification of ohmic, kinetic, and mass transport resistances within the cell, without introducing uncontrolled system level variables.^[^ ^68^ ^]^ In this setup, the electrolyte is first oxidized at the positive electrode and then circulated into the negative electrode, where it is reduced, and then is recirculated into the reservoir. An equivalent Randles‐like circuit with a Warburg element for convective diffusion (*W*_δ_) can be used to analyze the Nyquist plots obtained using EIS at open‐circuit potential to deconvolute ohmic, kinetic, and mass transport losses (inset of Figure 3c).^[^ ^16^, ^67^, ^68^ ^]^ In this circuit, *R*_Ω_ corresponds to ohmic resistance, *R*_CT_ to charge‐transfer resistance, and *R*_MT_ extracted from *W*_δ_ relates to mass‐transfer resistance. Using this framework, the product *iR*_Ω_ can be used to approximate ohmic contributions, which can be removed from polarization curve analysis to focus on kinetic and mass transport resistances (Figure 3b). We note that the resistances determined from the equivalent circuit model should be treated at most semiquantitatively, as the relatively simple model does not capture physical representations of the electrodes. We also used the single‐electrolyte setup under flow in supporting electrolyte (2 m HCl) to measure electrochemically accessible surface area (ECSA) representing the amount of available surface for double‐layer ion adsorption.

**Figure 3:**
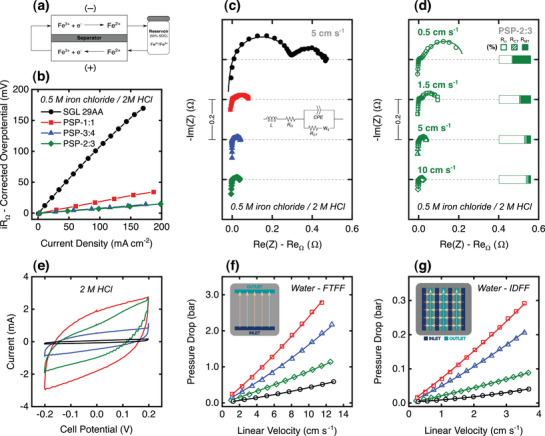
In situ single electrolyte performance. a) Schematic of iron chloride single electrolyte setup with a single reservoir. b) Polarization curves at 5 cm s^–1^ for SGL 29AA, PSP‐1:1, PSP‐3:4, and PSP‐2:3. c) Electrochemical impedance spectroscopy at 5 cm s^–1^ for SGL 29AA, PSP‐1:1, PSP‐3:4, and PSP‐2:3. Solid lines are fitted using the equivalent circuit shown in the inset. d) Electrochemical impedance spectroscopy for PSP‐2:3 at four different flow rates (0.5, 1.5, 5, and 10 cm s^–1^). Solid lines are fitted using the equivalent circuit shown in the inset. e) Capacitance measurements to determine electrochemically accessible surface area in 2 m HCl supporting electrolyte at a flow rate of 5 cm s^–1^ and 20 mV s^–1^ scan rate. A full range of scan rates can be found in Figure S11 (Supporting Information). Pressure drop with water as the fluid using a f) flow‐through flow field (FTFF) and g) interdigitated flow field (IDFF) for SGL29AA and PSP materials. Symbols are the data points, whereas lines are the fits using the Darcy–Forchheimer equation; values are summarized in Table S6 (Supporting Information).

Figure 3b shows the current density output at a given applied *iR*_Ω_‐corrected overpotential for the three different phase‐separated electrode samples (PSP‐1:1, PSP‐3:4, and PSP‐2:3) compared to a commercial SGL 29AA pristine electrode at an estimated linear velocity of 5 cm s^–1^. The average thicknesses of the synthesized electrodes were 670 ± 56 µm, whereas the average thickness of SGL 29AA was 186 ± 4 µm (Table S3, Supporting Information); a single electrode on each side was used for the single‐electrolyte iron chloride analysis. To account for differences and variability in electrode thicknesses, experiments were performed at equivalent superficial linear velocities, $v_{e}=\frac{Q}{t_{e}w_{e}}$, whereby *Q* is the volumetric flow rate (m^3^ s^–1^), *t*_e_ is the compressed electrode thickness (m), and *w*_e_ is the electrode width. In this study, we eschewed thermal pretreatments of the materials owing to their nuanced effects on the relevant electrode properties as the efficacy of such treatment is dependent on manufacturing routes and carbonization conditions idiosyncratic to each electrode.^[^ ^24^, ^69^, ^70^ ^]^ Thus, we decided to compare pristine SGL 29AA to pristine PSP electrodes, sacrificing power performance for clearer comparative analysis. Future work will explore the effect of well‐known electrode pretreatment strategies on PSP electrode performance. Regardless of the PAN:PVP ratio, all PSP electrodes exhibited lower polarization losses as compared to the SGL 29AA electrode in both single‐electrolyte iron chloride flow cell (Figure 3b) and full cell vanadium electrolyte (**Figure** **4**a) in this particular study. The performance of the PSP materials is consistent and reproducible for samples across different batches (Figure S8, Supporting Information). Furthermore, the volumetric ECSA measured in 2 m HCl supporting electrolyte for the PSP material sets exceeds that of the single SGL 29AA (1.12 × 10^5^ m^–1^); a representative capacitance measurement comparing the electrode set at 20 mV s^–1^ is shown in Figure 3e, whereas the full set of capacitance measurements can be found in Figure S11 (Supporting Information). For reference, the estimated values are of the same order of magnitude as for the cylindrical bundle of fibers approximation commonly used to estimate volumetric surface area (*a*), $a\;=\frac{4(1-\varepsilon )}{d_{f}}\;$. Specifically, for a material with porosity (ε) of 0.75 and fiber diameter (*d*_f_) of 8 µm, a volumetric surface area of 1.25 × 10^5^ m^–1^ would be attained.^[^ ^29^, ^71^, ^72^, ^73^ ^]^ The PSP‐1:1 showed the largest volumetric ECSA of all the electrodes (6.54 × 10^5^ m^–1^), followed by the PSP‐2:3 (3.66 × 10^5^ m^–1^) and the PSP‐3:4 (2.71 × 10^5^ m^–1^). The PSP materials all demonstrated higher ECSA than the SGL 29AA. The larger ECSA for the PSP‐1:1 is expected, as lower total porosity correlates with increased specific area assuming similar surface morphology. Interestingly, single‐electrolyte polarization and impedance demonstrated improved performance in lower surface area PSP‐3:4 and PSP‐2:3 compared to the higher surface area PSP‐1:1. As shown in Figure 2c, the PSP‐1:1 microstructure is sharply bimodal, divided into a predominantly macrovoid region and a region that is composed of smaller microvoids. We hypothesize that compared to the PSP‐2:3 and PSP‐3:4, the partition of macro‐ to microvoids leads to a less favorable microstructure for mass transport. Thus, we rationalize there is a discrepancy between the capacitance‐measured surface area and the utilizable surface area for electrochemical reactions. Nyquist plots shown in Figure 3c corroborate that larger mass transport resistance hinders PSP‐1:1 as compared to PSP‐3:4 and PSP‐2:3.

**Figure 4:**
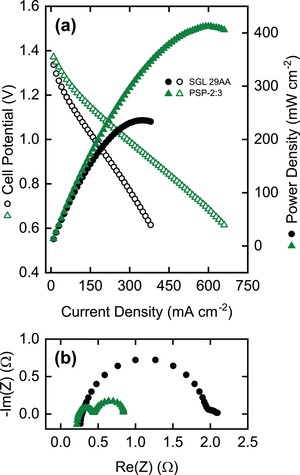
Full cell vanadium redox flow battery polarization and impedance. The electrolyte was 1.5 m V in 2.6 m H_2_SO_4_ supporting electrolyte. The linear velocity was 10 cm s^–1^. a) Discharge polarization with power density curves and b) electrochemical impedance spectroscopy for SGL 29AA (black circles) and PSP‐2:3 (green triangles).

To further investigate the effect of mass transport in the PSP electrodes, the electrolyte linear velocity was varied (Figure 3d). A breakdown of the relative contributions from ohmic, kinetic, and mass transport resistances is shown in the bar charts of Figure 3d. For the PSP‐2:3, at a low flow rate (0.5 cm s^–1^), mass transport overpotential is the most dominant source of loss, followed by ohmics and kinetics. As the flow rate increases, ohmics become increasingly more important, eventually comprising 80% of the overpotential by 5 cm s^–1^, and 86% by 10 cm s^–1^. Analogous Nyquist plots obtained using EIS as a function of flow rate for representative PSP‐3:4, PSP‐1:1, and SGL 29AA samples are shown in Figure S9 (Supporting Information), and a full summary of the resistances is provided in Table S5 (Supporting Information). In this equivalent circuit analysis, kinetic resistance remained a minor contributor until the highest velocity of 10 cm s^–1^, when convective flux enhances mass transport to the point where it is of similar magnitude to the kinetic rates. The mass transfer resistances also rapidly decrease and plateau with increasing linear velocity for all materials, although the mass transfer resistance for the PSP materials at 10 cm s^–1^ or greater linear velocities plateau at lower values than the SGL 29AA (Figure S10, Supporting Information). We note that the ohmic resistance of the PSP materials measured from the *x*‐intercept of the Nyquist plots ranged on average from 0.207 to 0.311 Ω, similar in magnitude to the average ohmic resistance of the pristine commercial material, 0.246 Ω (Table S4, Supporting Information). The results suggest that the lower carbonization temperatures used for our process did not hinder the effective conductivity of the electrodes. Using lower carbonization temperatures may eliminate the need for the higher graphitization temperature step, thus reducing manufacturing costs and improving economic viability for PSP electrodes.^[^ ^54^ ^]^ Finally, the pressure drop was determined as a function of linear velocity using both flow‐through flow fields (FTFF) and interdigitated flow fields (IDFF) to assess pumping losses, using water as the fluid (Figure 3f,g). In FTFF, SGL 29AA had the highest permeability (4.8 × 10^–11^ m^2^), followed by PSP‐2:3 (2.0 × 10^–11^ m^2^), PSP‐3:4 (1.1 × 10^–11^ m^2^), and PSP‐1:1 (7.6 × 10^–12^ m^2^). For IDFF, the same trend held, with SGL29AA (3.2 × 10^–10^ m^2^), PSP‐2:3 (7.5 × 10^–11^ m^2^), PSP‐3:4 (3.3 × 10^–11^ m^2^), and PSP‐1:1 (2.2 × 10^–11^ m^2^). The permeabilities measured for the PSP materials are comparable to other commercial carbon‐fiber‐based electrodes, which are generally in the range of ≈2.0–7.2 × 10^–11^ m^2^.^[^ ^16^ ^]^

Although the single‐electrolyte experiments encourage the use of PSP materials for convection‐driven applications, the PSP‐2:3 material appears the most promising of the set, as the higher porosity and thus, permeability, reduces pressure losses during operation (see pressure drop measurements in Figure 3f,g). We next investigated the performance of PSP electrodes in a vanadium redox flow battery (VRFB), which represents the current state‐of‐the‐art from lab to pilot scale and which is the most widely commercialized RFB technology.^[^ ^21^, ^74^, ^75^, ^76^, ^77^, ^78^ ^]^ For the same reasons previously discussed, SGL 29AA has been chosen as a commonly used fibrous material toward advanced reactor architectures with thinner electrode designs.^[^ ^17^, ^24^, ^33^, ^79^ ^]^ Discharge polarization and power density curves along with electrochemical impedance spectroscopy of vanadium redox flow cells at 50% SoC for the PSP‐2:3 and pristine SGL 29 AA carbon electrodes are shown in Figure 4a. Galvanostatic cycling of the PSP‐2:3 electrode is provided in the Supporting Information (Figure S12, Supporting Information). Encouragingly, the results demonstrate that PSP‐2:3 has significantly enhanced power density compared to the commercial pristine. Inspection of the Nyquist plots (Figure 4b) suggests that reduced kinetics and total resistance resulted in the enhanced power output, as the higher frequency semicircle which typically represents charge transfer kinetics was significantly reduced in magnitude for the PSP material. Additionally, the ohmic resistance of the PSP‐2:3 (0.235 Ω) was slightly lower compared to SGL 29AA (0.280 Ω) in the vanadium electrolyte. We attribute the bimodal pore size distribution of the PSP material to benefit full cell polarization, where microvoids provide high surface area thereby reducing kinetic resistance, and the presence of macrovoids provides channels that reduce convective resistance, allowing for more complete electrode utilization.

In summary, we show that NIPS‐derived electrodes consisting of interconnected, continuous porous networks hold promise for RFBs and offer a credible platform for materials innovation, posing compelling alternatives to current fiber‐based electrodes. The utilization of the NIPS technique as applied herein enables production of an unprecedented yet beneficial set of high porosity and multimodal materials. In addition to developing a versatile and potentially scalable method for fabricating designer RFB electrodes, we have systematically characterized the materials using spectroscopic techniques and evaluated their performance in a single‐electrolyte cell configuration, culminating in a full cell demonstration. Encouraging enhancement in power capabilities in both the iron chloride single electrolyte cell and the vanadium full cell motivates further exploration into the extensive NIPS parameter space. We posit that the combination of large macrovoid structures in tandem with high surface area microvoids mitigates convective transport and kinetic losses, respectively. We anticipate that optimization of the microstructural features beyond the examples chosen in this study can further enhance RFB performance. Furthermore, the interconnected porous networks produced from NIPS are inherently well‐suited for the design of electrodes with porosity gradients. Beyond RFB electrodes, the presented material concept may also be tailorable for a wide range of convection‐driven electrochemical technologies (e.g., fuel cells, electrolyzers) where local variations of pore size and porosity are necessary to optimize complex multiphase transport.

## Experimental Section

### Electrode Scaffold Formation

In this embodiment, the PVP and DMF dissolved into the coagulation bath upon submersion, leaving behind a porous PAN framework.^[^ ^57^, ^80^, ^81^ ^]^ Subsequent thermal stabilization and carbonization of the polymer scaffold led to the desired electrically conductive electrode. Samples were made by mixing the following amounts of PAN and PVP in 10 mL of DMF: 1 g of PAN to 1 g PVP (1:1 PAN:PVP by mass), 0.857 g of PAN to 1.143 g PVP (3:4 PAN:PVP by mass), or 0.8 g of PAN to 1.2 g PVP (3:4 PAN:PVP by mass). The powder and solvent were subsequently thoroughly mixed after heating in a 70 °C oil bath. An in‐house glass mold for casting the mixed polymer solution was constructed on an 18 × 18 cm^2^ glass plate using 5 × 7 cm^2^ notches having a depth of 1.1 mm (Figure S1, Supporting Information). Once cooled to room temperature, the polymer solution was poured in the notches, and the edge of a doctor blade was used to evenly cast the solution into the glass notches. After 10 min at room temperature, the casted solution was carefully immersed into a water bath (water level 6 cm above the casted solutions). Polymeric scaffolds were left to phase‐separate overnight at room temperature, after which they were transferred into a deionized (DI) water (Milli‐Q Millipore, 18.2 MΩ cm) bath and left overnight at 70 °C to remove the remaining PVP still present in the porous structure. Afterward, the polymeric scaffolds were dried between two paper sheets and placed between poly(tetrafluoroethylene) (PTFE, Teflon) plates in an oven at 80 °C for >4 h for drying. Each polymeric scaffold was compressed with 0.399 cm thick, 5.1 × 10.8 cm^2^ alumina ceramic blocks (McMaster‐Carr) weighing 100 g on top of the Teflon plates.

### Thermal Stabilization and Carbonization

Thermal stabilization of the PAN membranes was conducted to crosslink the polymer network and improve the final mechanical properties of the electrodes.^[^ ^52^, ^53^ ^]^ Membranes were sandwiched between two sheets of alumina paper (Profiltra B.V.) and two ceramic plates. Each membrane was compressed with 100 g on top of the ceramic plates during thermal stabilization. Membranes were thermally stabilized in air at 270 °C for 1 h at a ramp rate of 2 °C min^–1^. Directly following the thermal stabilization, membranes were sandwiched by the ceramic plates and placed in a tubular oven under a nitrogen flow of 2 L min^–1^. The carbonization sequence was: room temperature to 850 °C (ramp rate of 5 °C min^–1^), hold for 40 min, 850 to 1050 °C (ramp rate of 5 °C min^–1^), hold for 40 min, cool down to room temperature.^[^ ^29^ ^]^

### Ex Situ Characterization

A detailed description of the physicochemical characterization is provided in Section S3 (Supporting Information). The ex situ characterization techniques are briefly described in the information which follows.

### SEM

The microstructure and morphology of the prepared electrodes were analyzed by a JEOL JSM‐IT100 SEM at 10 kV acceleration voltage. Cross‐section sample preparation was performed by dipping the electrodes in a mixture of 50:50 (v/v) H_2_O/isopropyl alcohol prior to cryofracturing in liquid nitrogen. Noncarbonized samples were coated with gold in a JEOL JFC‐2300HR at 90 mA for 60 s for imaging.

### XTM

3D scans of the NIPS‐prepared electrode materials were collected using a Zeiss Xradia 620 Versa. The PSP‐2:3 sample was imaged with a 20× objective at a voltage of 50 kV, power of 4.5 W, 3.5 s exposure time, and 0.39 µm pixel size.

### MIP

Analysis of pore size distributions was performed using an AutoPore IV 9500 using ≈100 mg electrode samples and a 5 cm^3^ volume penetrometer. Pore diameters were calculated assuming a cylindrical shape and mercury–carbon contact angles of 130° (advancing and receding). The porosity of the bulk electrodes was estimated by registering the mass of the material before and after full imbibition with mercury, assuming a complete pore filling.

### Raman Spectroscopy

The molecular structural signature of the bulk carbonaceous materials was studied with a 300R confocal Raman microscope. The laser used for Raman was an UHTS300S_Green_NIR working at a wavelength of λ = 532.306 nm. The grating (G2) had a groove density of 600 gr mm^−1^ and a blaze wavelength (BLZ) of 500 nm. The spectral center was set at 2400 cm^−1^, and the integration time was set at 5 s. Every sample was analyzed using the laser power set at 4.822 mW and a total of 50 accumulations.

### XPS

Surface chemical functionalities were analyzed with a Thermo Scientific K‐Alpha equipped with a monochromatic small‐spot X‐ray source and a 180° double focusing hemispherical analyzer with a 128‐channel detector. Spectra were recorded with an aluminum anode (Al Kα = 1486.6 eV) operating at 72 W with a spot size of 400 µm in diameter. Survey scans were measured at a constant pass energy of 200 eV and region scans at 50 eV. The background pressure was 2 × 10^–9^ mbar, and the pressure used during measurements was 3 × 10^–7^ mbar (argon) because of the charge compensation dual beam source.

### Flow Cell Electrochemical Testing

For the single‐electrolyte experiments, iron(II) chloride tetrahydrate (FeCl_2_·4H_2_O, 98%, Sigma Aldrich), iron(III) chloride hexahydrate (FeCl_3_·6H_2_O, 97%, Sigma Aldrich), and hydrochloric acid (HCl, 37%, Sigma Aldrich) were dissolved in DI water. The total concentration of active species was 0.5 m at 50% state‐of‐charge, with 2 m HCl supporting electrolyte. Daramic 175 (175 µm thick, Daramic) microporous separator was used. FTFF was used for single‐electrolyte polarization, impedance, and capacitance measurements. Volumetric flow rates were adjusted based on electrode thickness (Table S3, Supporting Information) to match a range of linear velocities (0.5, 1.5, 5, and 10 cm s^–1^). A compression of ≈20% was used for all samples. The porous electrode capacitance was used to determine the volumetric ECSA of the electrodes. Specifically, cyclic voltammetry was performed in the single‐electrolyte configuration in 2 m HCl supporting electrolyte across the range of −0.2 to 0.2 V to avoid Faradaic reactions, at an electrolyte velocity of 5 cm s^–1^ at scan rates of 20, 50, 100, 150, and 200 mV s^–1^. The capacitance was determined from $i\;=\;C\frac{dV}{dt}$, where *i* is the average of the positive and negative current at 0 V (A), *C* represents the capacitance (F), and d*V*/d*t* is the scan rate (V s^–1^). The specific capacitance of glassy carbon (23 µF cm^–2^) was used as a reference to estimate the porous electrode ECSA.^[^ ^24^, ^82^ ^]^ To account for differences in electrode thicknesses, the ECSA was normalized by the volume occupied by the compressed electrode, leading to a volumetric ECSA. Pressure drop measurements were conducted using water as the working fluid, taking the difference of the inlet and outlet measurements averaged over 20 s at multiple flow rates.

For discharge polarization and impedance measurements in the full‐cell configuration VRFB, starting electrolytes were composed of 1.5 m VOSO_4_ in 2.6 m H_2_SO_4_. 50% SoC electrolyte was prepared via electrolysis as described in detail in a previous report.^[^ ^27^ ^]^ A Nafion 212 membrane (50.8 µm nominal thickness, Fuel Cell Store) was presoaked >24 h in 2.6 m H_2_SO_4_ at ambient temperature prior to use as a separator. FTFF was used in combination with the electrode at a linear velocity of 10 cm s^–1^ for discharge polarization and impedance measurements. All iron chloride single‐electrolyte experiments were carried out with single electrodes. Full vanadium cells were performed with a stack of 3× SGL 29AA electrodes to match the PSP electrodes thickness. More in‐depth details are provided in Section S4 (Supporting Information).

## Conflict of Interest

The authors declare no conflict of interest.

## Author Contributions

C.T.‐C.W. and R.R.J. contributed equally to this work. C.T.‐C.W. contributed to the conceptualization, methodology, validation, formal analysis, investigation, data curation, writing—original draft, writing—review and editing, and visualization. R.R.J. contributed to the conceptualization, methodology, validation, formal analysis, investigation, data curation, writing—original draft, writing—review and editing, and visualization. Y.‐M.C. contributed to resources. K.N. contributed to funding, resources, writing—review and editing, and supervision. F.R.B. contributed to funding, resources, writing—review and editing, and supervision. A.F.‐C. contributed to conceptualization, methodology, investigation, funding, resources, writing—original draft, writing—review and editing, project administration, and supervision.

## Supporting information

Supporting Information
