# Supplementary material for: Non‐Solvent Induced Phase Separation Enables Designer Redox Flow Battery Electrodes
Source: Adv Mater. 2021 Mar 2;33(16):2006716. doi: 10.1002/adma.202006716 (PMC9290313; doi:10.1002/adma.202006716)
Supplement: Supplementary file 1 — Supporting Information [file ADMA-33-2006716-s001.pdf]

© 2021 Wiley-VCH GmbH

ADVANCED MATERIALS

Supporting Information

for *Adv. Mater.,* DOI: 10.1002/adma.202006716

Non-Solvent Induced Phase Separation Enables Designer Redox Flow Battery Electrodes

*Charles Tai-Chieh Wan, Rémy Richard Jacquemond, Yet-Ming Chiang, Kitty Nijmeijer, Fikile R. Brushett, and Antoni Forner-Cuenca**

Supporting Information

Non-Solvent Induced Phase Separation as a Paradigm for Designer Redox Flow Battery Electrodes

*Charles Tai-Chieh Wan[#], Rémy Richard Jacquemond[#], Yet Ming-Chiang, Kitty Nijmeijer, Fikile R. Brushett, Antoni Forner-Cuenca**

[#] Co-first authorship

Corresponding author: a.forner.cuenca@tue.nl

Section S1 – Materials and chemicals 2

Section S2 - Synthesis of phase separated electrodes 3

Scaffold formation - 3

Thermal stabilization - 4

Carbonization - 4

Section S3 – Imaging and physicochemical characterization methods 5

Scanning electron microscopy - 5

Pore size extraction from SEM micrographs - 5

X-Ray Tomography - 7

Mercury intrusion porosimetry - 9

X-ray photoelectron spectroscopy: 12

Raman spectroscopy – 14

Section S4 – In situ electrochemical characterization 15

Flow cell experiments: 15

Linear electrode velocity conversions - 16

Averaged area-specific ohmic resistance extracted from impedance spectroscopy - 17

Impedance fitting tables - 18

Additional EIS plots as a function of flow rate - 20

Extracted mass transfer resistance from Nyquist plot fittings - 21

Capacitance measurements - 22

Pressure drop measurements for interdigitated and flow through flow fields - 23

Full vanadium redox flow battery cycling experiments – 25

Section S1 – Materials and chemicals

Isopropanol (IPA ACS reagent ≥ 99.5%, Sigma Aldrich). Dimethyl formamide (DMF for HPLC 99.9%, Sigma Aldrich). Polyvinylpyrrolidone (PVP Average Mw ~ 1,300,000 by LS, Sigma Aldrich). Polyacrylonitrile (PAN Average Mw ~ 150,000, Sigma Aldrich). Hydrochloric acid (HCl ACS reagent 37%, Sigma Aldrich). Iron (II) chloride tetrahydrate ($FeCl_2$•$4H_2O$ 99.0%, Sigma Aldrich). Iron (III) chloride hexahydrate ($FeCl_3$•$6H_2O$ 99%, Sigma Aldrich). MiliQ water 18 MΩ was used for every experiment. Benchmark commercial electrode material Sigracet 29AA (nominal thickness ~ 190 µm thick, Fuel Cell Store) was used without any further treatment. A microporous separator Daramic® 175 (nominal thickness ~175 µm, Daramic®) was used in iron chloride single-electrolyte experiments, while Nafion 212 (nominal thickness ~ 50.8 µm, Fuel Cell Store) was used in vanadium flow cell testing.

A 50% state of charge (SoC) 0.5 M iron solution in 2 M HCl was prepared by dissolving 12.42 g of $FeCl_2$•H_2O and 16.89 g of $FeCl_3$•$6H_2O$ in 41.75 mL of HCl, finally water was added to reach a total volume of 250 mL.

Section S2 - Synthesis of phase separated electrodes

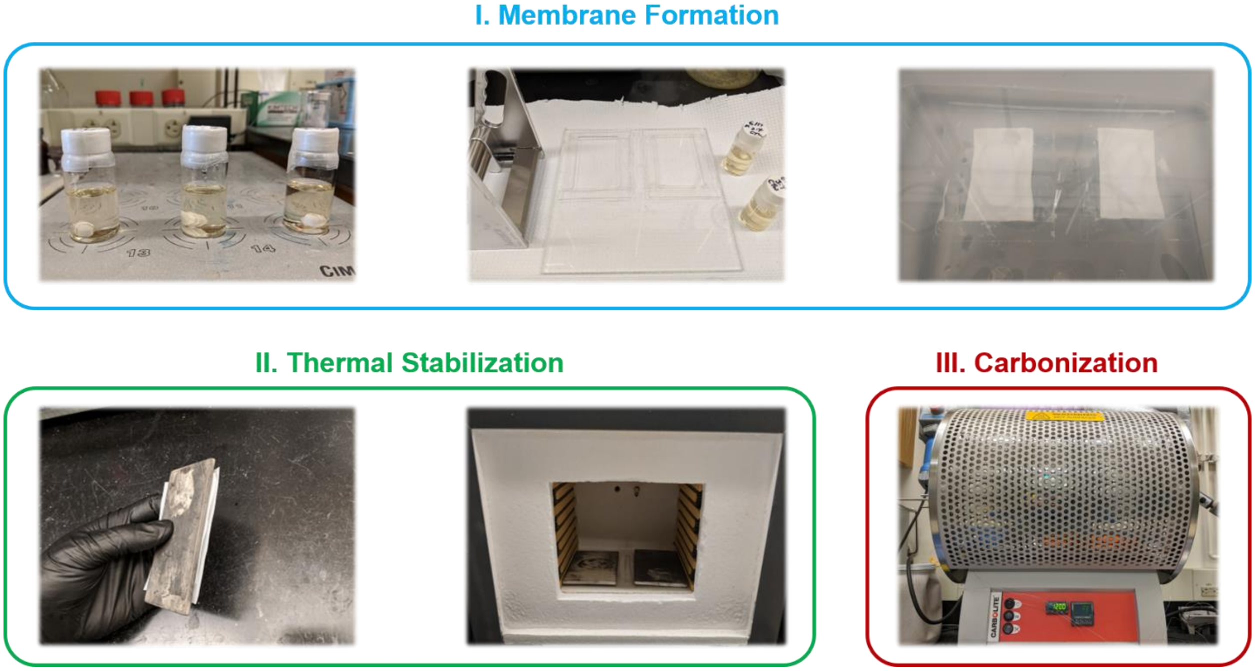

Figure S1: *Photographs of the preparation stages in the phase separation process from start to finish, specifically - I. membrane formation, II. thermal stabilization, and III. carbonization.*

Scaffold formation -

Porous carbon electrodes were prepared by phase separation of polymer precursors in a coagulation bath of non-solvent (water) and subsequent carbonization. PAN and PVP powders were combined in scintillation vials, followed by the addition of the DMF solvent. The powder and solvent was subsequently fully mixed after heating in a 70°C oil bath. An in-house glass mold for casting the mixed polymer solution was constructed on an 18 × 18 cm^2 glass plate using 5 × 7 cm^2 notches having a depth of 1.1 mm (Figure S1). Once cooled to room temperature, the polymer solution was poured in the notches, and the edge of a doctor blade was used to evenly cast the solution into the glass notches. After 10 min at room temperature, the casted solution was carefully immersed into a water bath (water level 6 cm above the casted solutions). Polymeric scaffolds able to phase separate overnight at room temperature, after which they were transferred into a water (Milli-Q, Millipore 18.2 MΩ cm) bath and left overnight at 70°C to remove the remaining PVP still present in the porous structure. Afterwards, the polymeric scaffolds were dried between two paper sheets and placed between Teflon plates in an oven at 80°C for > 4 h for drying. Each polymeric scaffold was compressed with 0.157 in. thick, 2 in. × 4-1/4 in. alumina ceramic blocks (McMaster-Carr) weighing 100 g on top of the Teflon plates.

Thermal stabilization -

Thermal stabilization of the PAN membranes was conducted to crosslink the polymer network and improve the final mechanical properties of the electrodes.[1,2] Membranes were sandwiched between two sheets of alumina paper (Profiltra B.V.) and two ceramic plates. Each membrane was compressed with 100 g on top of the ceramic plates during thermal stabilization. Membranes were thermally stabilized in air at 270°C for 1 h at a ramp rate of 2°C min^{-1}.

Carbonization -

Directly following the thermal stabilization, membranes were sandwiched by the ceramic plates and placed in a tubular oven under a nitrogen flow of 2 L min^{-1}. The carbonization sequence was: room temperature to 850°C (ramp rate of 5°C min^{-1}), hold for 40 min, 850°C to 1050°C (ramp rate of 5°C min^{-1}), hold for 40 min, cool down to room temperature.[3] Carbonization yields are shown in Figure S2.

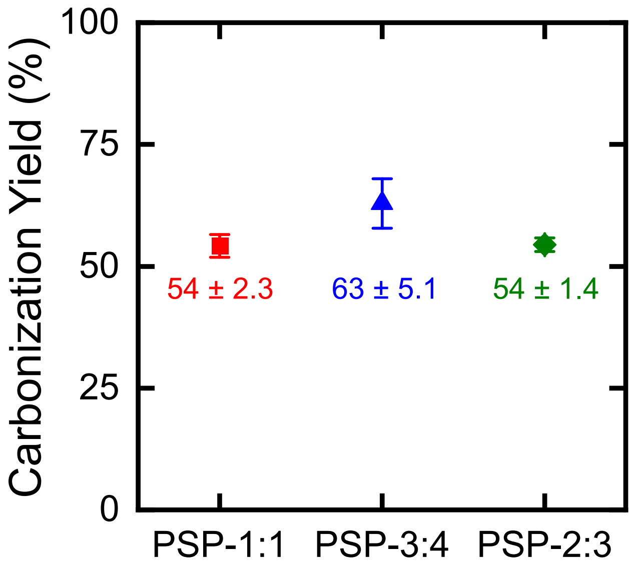

Figure S2: *Percent yield calculated as the remaining mass after carbonization divided by thermally stabilized mass for PSP-1:1, PSP-3:4, and PSP-2:3. All experiments were run in triplicate.*

Section S3 – Imaging and physicochemical characterization methods

Scanning electron microscopy -

The microstructure and morphology of the prepared electrodes was analyzed by a JEOL JSM-IT100 scanning electron microscope (SEM) at 10kV acceleration. Cross-section sample preparation was performed by dipping the electrodes in a mixture of 50:50 (v/v) H_2O/Isopropanol prior to cryofracturing in liquid nitrogen. Non-carbonized samples were coated with gold in a JEOL JFC-2300HR at 90 mA for 60 s.

Pore size extraction from SEM micrographs -

Scanning electron micrographs were analyzed using ImageJ software. Each image prior to pore size extraction was optimized in terms of contrast and brightness in order to maximize the accuracy of the extraction. Macrovoids were extracted using the "Cell Outliner" plugin of ImageJ which targets areas where there is a steep change in brightness (Figure S3a). Microvoids were extracted in a similar fashion, in which the contrast and brightness of the original picture was optimized to obtain the best contrast between the pores and the electrode scaffold (Figure S3b). Finally, for both images, the "Analyze Particles" tool was employed to extract the pore size distribution in pixels which was subsequently converted to metric units by using the SEM magnification as the converting factor (Figure S3c).

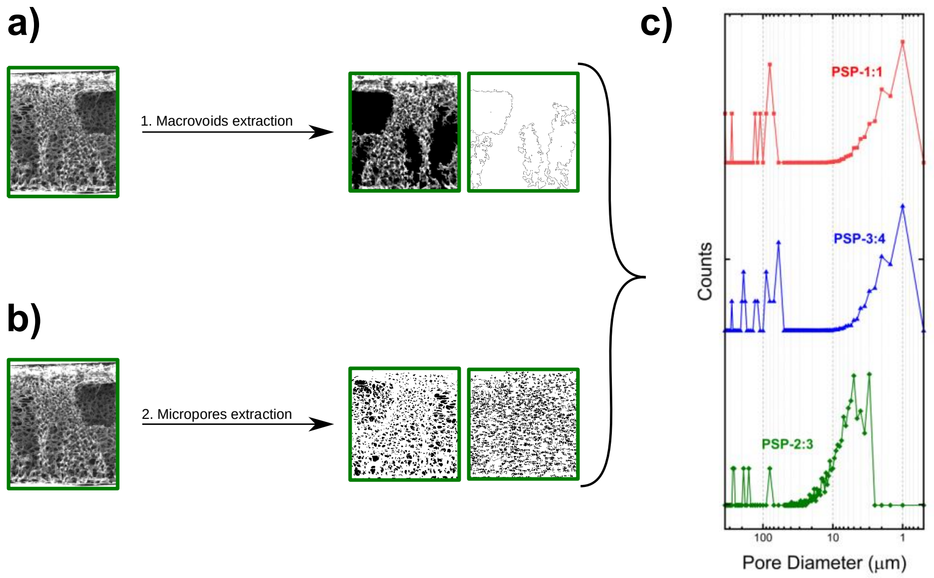

Figure S3: *Pore size distribution extraction procedure from SEM micrograph. a) Isolation of macrovoids using ImageJ cell outliner plugin for PSP-2:3. b) Extraction of microvoids using ImageJ built-in analyze particles tool for PSP-2:3. c) Pore size distribution extraction using ImageJ software, a multiplying factor has been applied to the macrovoid region (> 50 μm) to compensate for the difference in pores density relative to microvoids. Pore size distributions of the PSP materials are shown.*

We note that the pore size extraction was conducted assuming spherical pores. While this is a fair approximation for microvoids, it is less accurate for the highly irregularly shaped macrovoids. Another bias comes from the interference of background microvoids which are difficult to excise and can convolute estimation of foreground microvoid diameters. Pore size distribution (PSD) extraction from SEM micrographs is only intended to provide qualitative data on the PSD of the sample due to the aforementioned biases and to the loss of information when representing the PSD of a 3D sample by extracting a 2D cross-section. Nevertheless, when compared to other characterization techniques, SEM extraction provides a fast and qualitative analysis of the PSD which can be used as a coarse diagnostic tool.

The same method was applied to extract the PSD as a function of the electrode thickness, as shown in Figure S4. The SEM picture was first segmented into 8 sections of equal thicknesses prior to being analyzed in ImageJ using the procedure stated in the previous subsection.

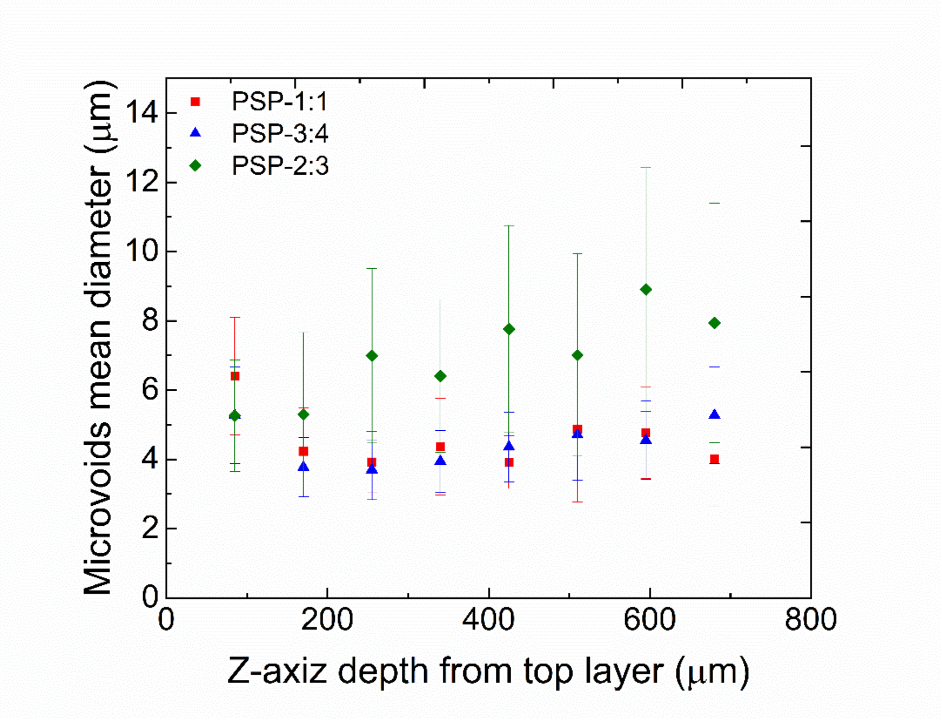

Figure S4: *Average microvoid diameter as a function of the z-depth position from the electrode top layer. A single cross-section was used for each sample. Multiple pores were used to calculate the average and standard deviation of the microvoids diameters 400 < n < 600 for each point.*

X-Ray Tomography -

Imaging of the phase separated materials were collected using a Zeiss Xradia 620 Versa. The PSP-1:1 sample was collected with a 4× objective at a voltage of 40 kV, power of 3 W, 20 s exposure time, and 0.87 μm pixel size. The PSP-3:4 sample was collected a 20× objective at a voltage of 40 kV, power of 3 W, 4 s exposure time, and 0.35 μm pixel size. The PSP-2:3 sample was collected a 20× objective at a voltage of 50 kV, power of 4.5 W, 3.5 s exposure time, and 0.39 μm pixel size. The settings were chosen to optimize image collection time without sacrificing resolution while also obtaining the largest possible sample volume. Resulting XTMs were segmented using the ImageJ Plugin Weka Segmentation.[4] The images and cross-sections were rendered using Dragonfly Pro software (Figure S4**)**.

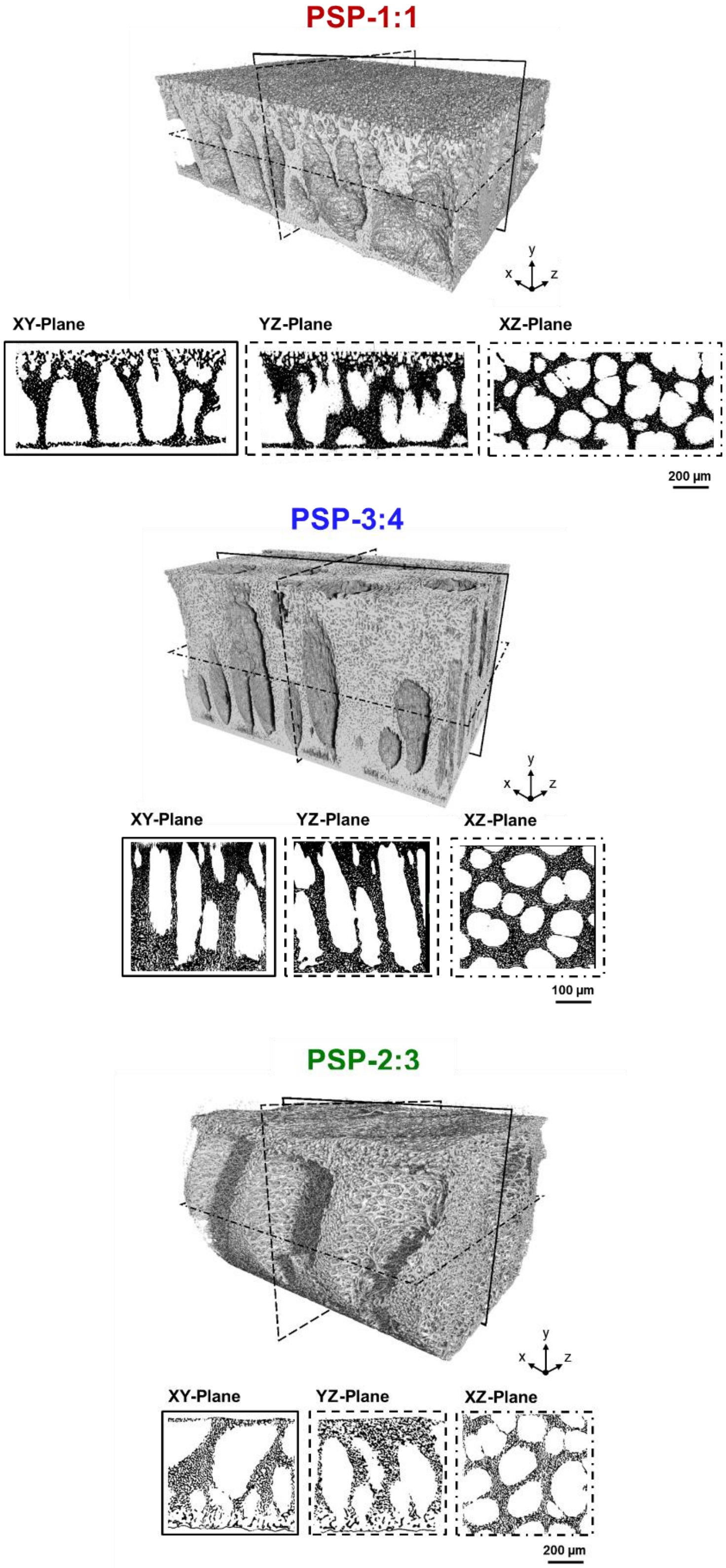

Figure S5: *Reconstructed 3D renderings of the PSP-1:1, PSP-3:4, and the PSP-2:3 from X-ray computed tomography. Select representative cross sections of the materials are given below the renderings.*

Mercury intrusion porosimetry -

The microstructure of the different porous electrodes was characterized using mercury intrusion porosimetry (MIP). The main assumption of the technique is that mercury is a non-wetting fluid and that the pores are perfect cylinders of a certain radius[5]. Note that MIP is a semi-quantitative technique to extract the PSD, the measurement relies on the assessment of permeability and mass transport of mercury in a porous sample and some architectural variations within a sample might not be accessible with this technique.

MIP analyses were performed using an AutoPore IV 9500 employing ca. 100 mg electrode sample and a 5 cm^3 volume penetrometer. Pore diameters were calculated assuming a cylindrical shape and mercury-carbon contact angles of 130° (advancing and receding) to determine the PSD. The porosity of the bulk electrodes was estimated by registering the mass of the material before and after full imbibition with mercury, assuming a complete pore filling. A full list of the obtained parameters is presented in Table S1.

Table S1 – *Microstructural of the studied electrodes extracted from MIP.*

	SGL	PSP-1:1	PSP-3:4	PSP-2:3
**Peak 1 fitting**				
**x_c**	58.8	9.5	55.4	36.1
**A**	222.8	23.5	18.7	21.0
**w**	27.1	3.5	34.4	24.4
**Skewness**	3.9	4.4	3.2	3.0
**Peak 2 fitting**				
**x_c**	1.4	2.8	33.9	15.0
**A**	0.9	6.0	18.4	58.2
**w**	1.1	1.2	18.4	6.7
**Skewness**	3.9	4.2	3.5	4.0
**Peak 3 fitting**				
**x_c**			20.3	8.3
**A**			36.0	3.1
**w**			16.4	2.1
**Skewness**			2.6	5.5
**Peak 4 fitting**				
**x_c**			12.0	3.8
**A**			65.2	7.6
**w**			5.2	1.6
**Skewness**			4.1	4.2
**Peak 5 fitting**				
**x_c**			8.4	
**A**			11.5	
**w**			2.5	
**Skewness**			5.1	
**Peak 6 fitting**				
**x_c**			4.6	
**A**			0.5	
**w**			0.9	
**Skewness**			6.4	
**Fit R^2**	0.95	0.98	0.88	0.98

A multi peaks fitting was performed in Origin software to extract the contribution of the different pore sizes on the overall PSD of each sample. A multi peaks gaussian fit was used and the parameters used for the fitting are given in table S1 with x_c being the centroid of the peaks, A is proportional to the peak area and w is proportional to the width of the peak. The skewness of each fitted peak was approach using the following equation:

$$Skewness = \frac{n}{(n-1)(n-2)} \sum_{i=1}^{n} (\frac{\bar{x_i} - \bar{x}}{sd})^3$$

The quality of the fit is displayed in Table S1 as the R square coefficient (R^2).

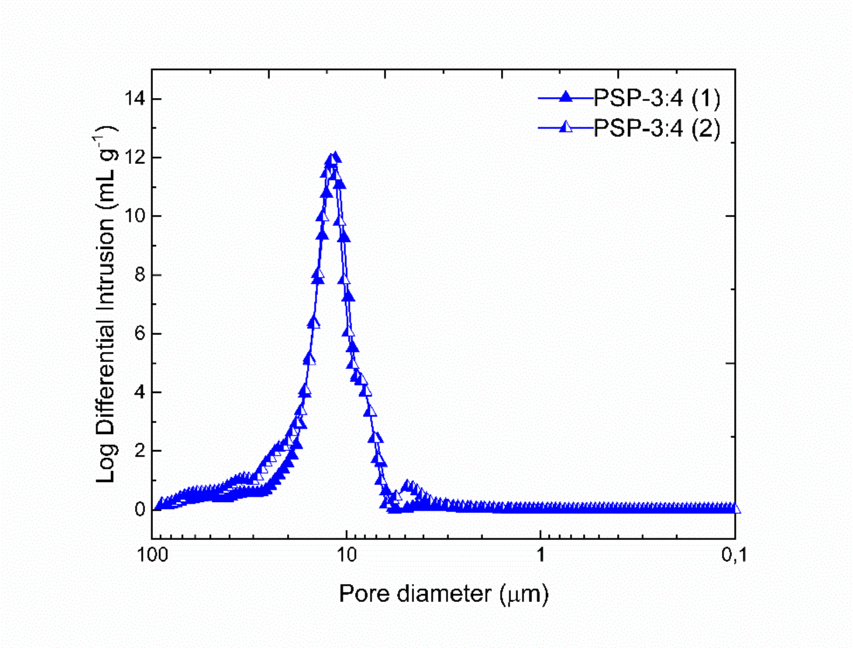

Figure S6: *Pore size distribution extracted from MIP measurements of two different PSP-3:4 samples used for MIP reproducibility assessment. Individual samples are denoted by different markers*

X-ray photoelectron spectroscopy:

Surface chemical functionalities were analyzed by X-ray photoelectron spectroscopy (XPS) with a Thermo Scientific K-Alpha equipped with a monochromatic small-spot X-ray source and a 180° double focusing hemispherical analyzer with a 128-channel detector. Spectra were recorded with an aluminum anode (Al Ka = 1486.6 eV) operating at 72 W with a spot size of 400 μm in diameter. Survey scans were measured at a constant pass energy of 200 eV and region scans at 50 eV. The background pressure was $2 \cdot 10^{-9}$ mbar and the pressure used during measurements was $3 \cdot 10^{-7}$ mbar (Argon) because of the charge compensation dual beam source.

C1s, N1s and O1s high-resolution spectra were analyzed using CasaXPS software. The fitting was done using a Shirley background and the deconvolution of the different contributions was calculated using Gaussian/Lorentzian shape lines with full width at half maximum values constrained at 2eV. The fitting was iterated until the residual standard deviation (STD) was close to unity (values between 1 and 2). The proposed peaks assignment is based on published XPS interpretations for similar types of carbonaceous materials.[6–9]

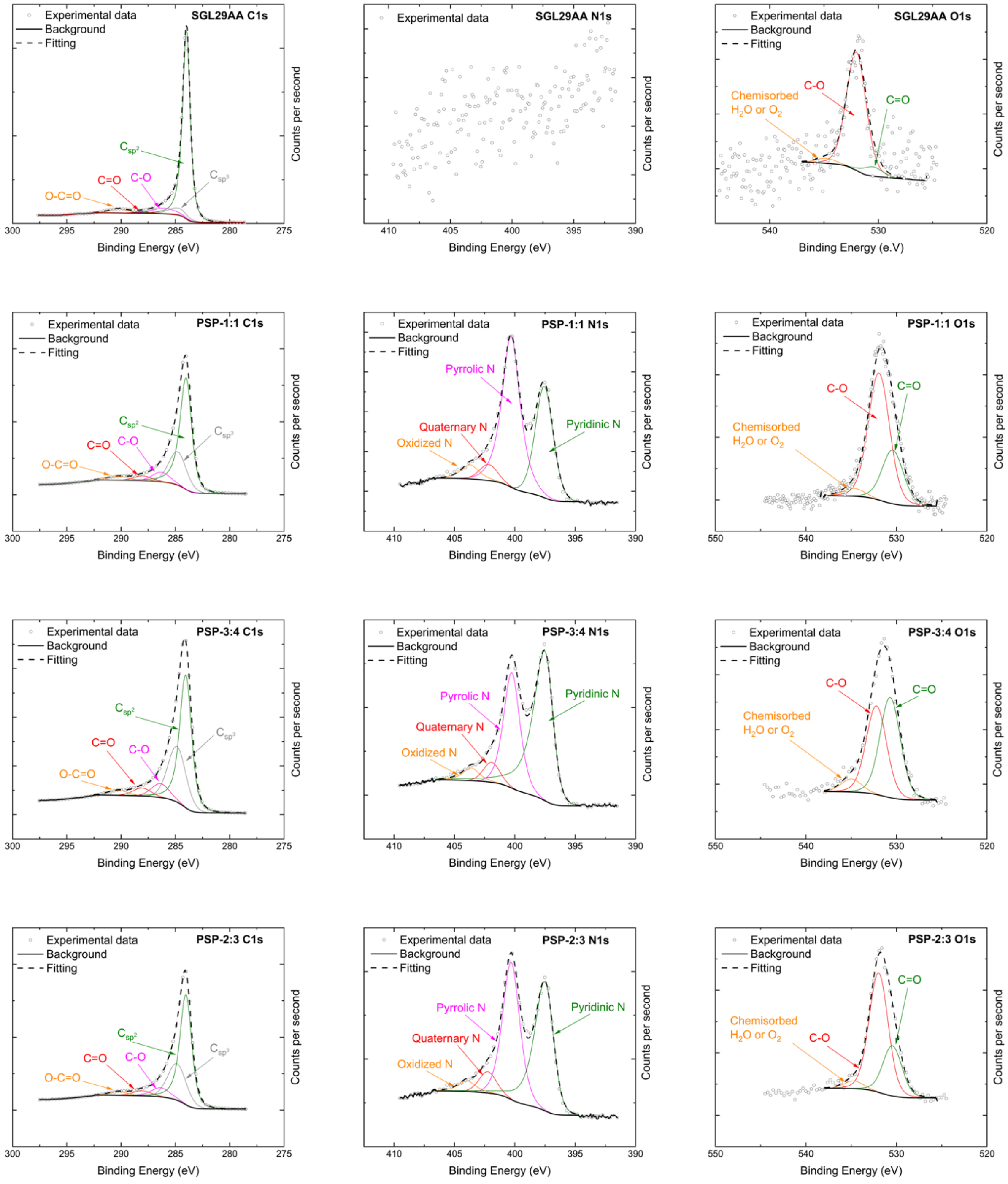

Figure S7: *C1s, N1s and O1s high resolution spectra with proposed peaks fitting for the pristine SGL29AA and the phase separated electrodes.*

Raman spectroscopy –

The molecular morphology of the bulk carbonaceous materials was studied with a 300R confocal Raman microscope. The laser used for Raman was a UHTS300S_Green_NIR working at a wavelength of λ = 532.306 nm. The grating (G2) had a groove density of 600 gr/mm and a blaze wavelength (BLZ) of 500 nm. The spectral center was set at 2400 cm^{-1} and the integration time was set at 5 s, every sample was analyzed using the laser power set at 4.822 mW and a total of 50 accumulations.

Table S2 – *Surface composition and morphology of all samples measured by XPS and Raman spectroscopy, respectively.*

	SGL 29AA	PSP-1:1	PSP-3:4	PSP-2:3
**Mean Nitrogen content (%at) (N=3)**	0.0 ± 0.0	7.8 ± 1.0	8.1 ± 1.9	11.9 ± 2.8
**Mean Oxygen content (%at) (N=3)**	0.6 ± 0.3	2.9 ± 1.0	3.1 ± 1.1	2.9 ± 0.3
**I_D/I_G (N=1 or 2)**	0.93	0.91 ± 0.02	0.94 ± 0.01	0.97 ± 0.03

Section S4 – In situ electrochemical characterization

Flow cell experiments:

The single electrolyte configuration is shown in **Figure 3**a. A 50% SoC electrolyte solution was circulated through the flow cell, leading to oxidation reaction at the anode and reduction at the cathode, maintaining constant SoC throughout the experiment. The RFB architecture included flow-through flow fields machined on impregnated graphite (G347B graphite, MWI Inc.), polypropylene flow diffusers (Adaptive Engineering), and Daramic® 175 (175 μm thick, Daramic®) microporous separator in between the positive and negative electrodes. Polytetrafluoroethylene (PTFE) gaskets were used for sealing the cell, and a compression of ca. 75-80% was used for all experiments. The dimensions of the active area of the electrodes was 1.5 cm × 1.7 cm, leading to a geometric area of 2.55 cm^2. During operation, 50 mL of the electrolyte solution was stored in a sealed glass vial connected to the RFB cell through Neoprene L/S 16 precision tubing (Cole Parmer). A peristaltic pump (Masterflex L/S series) was used to flush the electrolyte solution through the system. All the cells were pre-conditioned by flowing electrolyte through the electrodes at 10 mL min^{-1} for 10 min before data collection.

Polarization measurements were recorded with a Biologic VMP3 potentiostat between 0 and 0.6 V by potentiostatic hold of 30 s every 25 mV recording one data point per second. The average current and voltage were calculated from the last 50% collected points to approximate the steady state conditions. Electrochemical impedance spectroscopy (EIS) was performed using the same potentiostat at open circuit voltage (OCV), with an amplitude of 10 mV with a frequency range from 200 kHz to 10 mHz, averaging 6 points per decade. The equivalent circuit fitting was performed using the EC-Lab® ZFit program, from which the ohmic, kinetic, and mass transport resistances were determined.

For the single cell vanadium RFB, the same cell architecture and setup previously described above was used. A Nafion 212 membrane (50.8 μm nominal thickness, Fuel Cell Store) pre-soaked in 2.6 M H_2SO_4 for >24 h was used to separate the positive and negative electrolytes. Electrolyte solutions were electrochemical generated as described in previous reports.[7,10] Electrolyte solutions were continually purged with humidified nitrogen gas throughout the entirety of the experiments. For the discharge polarization, the starting solution was 50mL of 1.5 M $VOSO_4$ in 2.6 M H_2SO_4 for each reservoir with flow-through flow fields at 50% SOC. Following discharge polarization, EIS was performed at open circuit potential at 50% SOC to identify the kinetic, ohmic, and mass transport contributions to resistance in the full flow cell. The applied frequency ranged from 1 MHz to 10 Hz. 6 points per decade and an average of 5 measures per frequency were recorded. The sine amplitude was 10 mV. Galvanostatic cycling was performed to assess the stability of the phase separated electrodes with starting solutions of 15 mL of 1 M $VOSO_4$ in 3 M H_2SO_4 in each reservoir with interdigitated flow fields. The current density applied was 100 mA cm^{-2} for both charge and discharge. Cutoff voltages of 0.8 and 1.7 V for discharge and charge, respectively, were chosen to minimize side reactions.

Linear electrode velocity conversions -

To account for sample to sample variability in electrode thicknesses, performance was compared at equivalent superficial linear velocities (v_e), given by the following equation for a flow-through-flow-field:

$$v_e = \frac{Q}{t_e w_e}$$

where Q is the volumetric flow rate ($m^3\ s^{-1}$), t_e is the compressed electrode thickness (m), and w_e is the electrode width (0.015 m in all cases here). The electrode compression for all cells was ca. 75 – 80%. For the electrodes in this study, this corresponds to volumetric flow rates ranging from ~2 - 50 mL min^{-1}. The average thickness for the PSP materials is shown in **Table S1** and exhibited a relatively uniform range centered at ca. 667 µm. Given the reactor and electrode dimensions, the volumetric flow rates for the phase separated materials at superficial velocities of 0.5, 1.5, 5, and 10 cm s^{-1} were ca. 2.4, 7.2, 21, and 48 mL min^{-1}, with sample to sample variation. In the iron chloride symmetric cell, a single SGL 29AA electrode was used on both the positive and negative side to avoid complications from stacking multiple electrodes. Since the SGL 29AA electrode is relatively thin, the low volumetric flow rates result in slow peristaltic pumping and noisy data at low superficial velocities; thus, a higher range of superficial velocities was performed, specifically 1.5, 5, 10, and 20 cm s^{-1}, corresponding to volumetric flow rates of ca. 2.0, 6.7, 13, and 27 mL min^{-1}. For the full cell vanadium test, three SGL electrodes were stacked on each side in order to more closely match electrode thicknesses for a practical cell performance comparison, and thus the volumetric flow rates are simply multiplied by a factor of three.

Table S3 – Single electrode thickness for SGL 29AA and the phase separated materials.

	**SGL 29AA**	**PSP-1:1**	**PSP-3:4**	**PSP-2:3**
Thickness (µm)	186 ± 4	691 ± 47	667 ± 66	647 ± 49

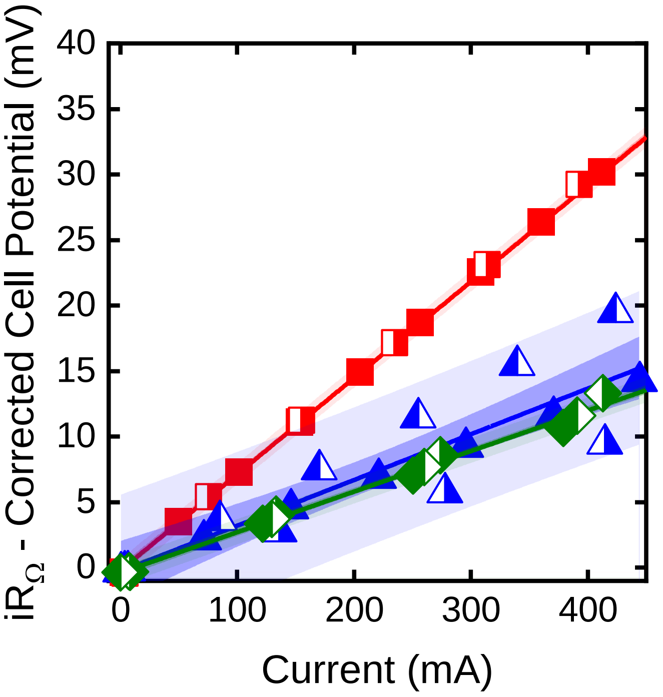

Figure S8: *Polarization curves at 5 cm s^{-1}. Inner shaded region is 95% confidence bound, while outer shaded region is the 95% prediction range for PSP-1:1 (red square), PSP-3:4 (blue triangle), and PSP-2:3 (green rhombus). Individual samples are denoted by different marker symbol. Experiments were repeated two or more times.*

Averaged area-specific ohmic resistance extracted from impedance spectroscopy -

Table S4 – *Summary of area-specific ohmic resistance extracted from EIS in iron chloride single electrolyte experiments.*

	**SGL 29AA**	**PSP-1:1**	**PSP-3:4**	**PSP-2:3**
Area-specific Ohmic Resistance (Ω cm^2)	0.627 ± 0.144	0.792 ± 0.216	0.720 ± 0.264	0.529 ± 0.230

Impedance fitting tables -

Table S5 – *Fitted parameters from electrochemical impedance spectroscopy at open circuit voltage for four separate electrodes at various representative linear velocities. Percentages refer to the relative contribution to the total resistance as determined by the equivalent circuit fitting.*

**Linear velocity ($cm\ s^{-1}$)**	**Area-specific total resistance ($\Omega\ cm^2$)**	**Area-specific ohmic resistance ($\Omega\ cm^2$)**	**Area-specific charge transfer resistance ($\Omega\ cm^2$)**	**Area-specific mass transfer resistance ($\Omega\ cm^2$)**
0.5	1.58	0.614 / 39%	0.161 / 10%	0.801 / 51%
1	1.19	0.607 / 51%	0.145 / 12%	0.439 / 37%
1.5	1.08	0.607 / 56%	0.160 / 15%	0.312 / 29%
5	0.837	0.591 / 71%	0.060 / 7.2%	0.186 / 22%
10	0.747	0.606 / 81%	0.069 / 9.3%	0.072 / 9.6%

PSP-1:1

PSP-3:4

**Linear velocity ($cm\ s^{-1}$)**	**Area-specific total resistance ($\Omega\ cm^2$)**	**Area-specific ohmic resistance ($\Omega\ cm^2$)**	**Area-specific charge transfer resistance ($\Omega\ cm^2$)**	**Area-specific mass transfer resistance ($\Omega\ cm^2$)**
0.5	1.459	0.760 / 52%	0.015 / 1.0%	0.684 / 47%
1	1.15	0.765 / 66%	0.015 / 1.3%	0.371 / 32%
1.5	1.04	0.768 / 74%	0.019 / 1.9%	0.252 / 24.2%
5	0.875	0.770 / 88%	0.029 / 3.3%	0.076 / 8.7%
10	0.837	0.768 / 92%	0.036 / 4.3%	0.033 / 4.0%

PSP-2:3

Linear velocity ($cm\ s^{-1}$)	Area-specific total resistance ($\Omega\ cm^2$)	Area-specific ohmic resistance ($\Omega\ cm^2$)	Area-specific charge transfer resistance ($\Omega\ cm^2$)	Area-specific mass transfer resistance ($\Omega\ cm^2$)
0.5	0.993	0.405 / 41%	0.024 / 2.4%	0.564 / 57%
1	0.745	0.391 / 53%	0.050 / 6.8%	0.304 / 41%
1.5	0.658	0.420 / 64%	0.031 / 4.7%	0.207 / 32%
5	0.520	0.415 / 80%	0.040 / 7.6%	0.064 / 12%
10	0.481	0.413 / 86%	0.035 / 7.2%	0.034 / 7.0%

Linear velocity ($cm\ s^{-1}$)	Area-specific total resistance ($\Omega\ cm^2$)	Area-specific ohmic resistance ($\Omega\ cm^2$)	Area-specific charge transfer resistance ($\Omega\ cm^2$)	Area-specific mass transfer resistance ($\Omega\ cm^2$)
0.5	-	-	-	-
1	-	-	-	-
1.5	2.50	0.976 / 39%	0.841 / 34%	0.682 / 27%
5	2.19	0.975 / 44%	0.833 / 38%	0.386 / 18%
10	2.08	1.02 / 49%	0.773 / 37%	0.290 / 14%
20	2.01	0.995 / 49%	0.801 / 40%	0.215 / 11%

SGL 29AA Paper

Additional EIS plots as a function of flow rate -

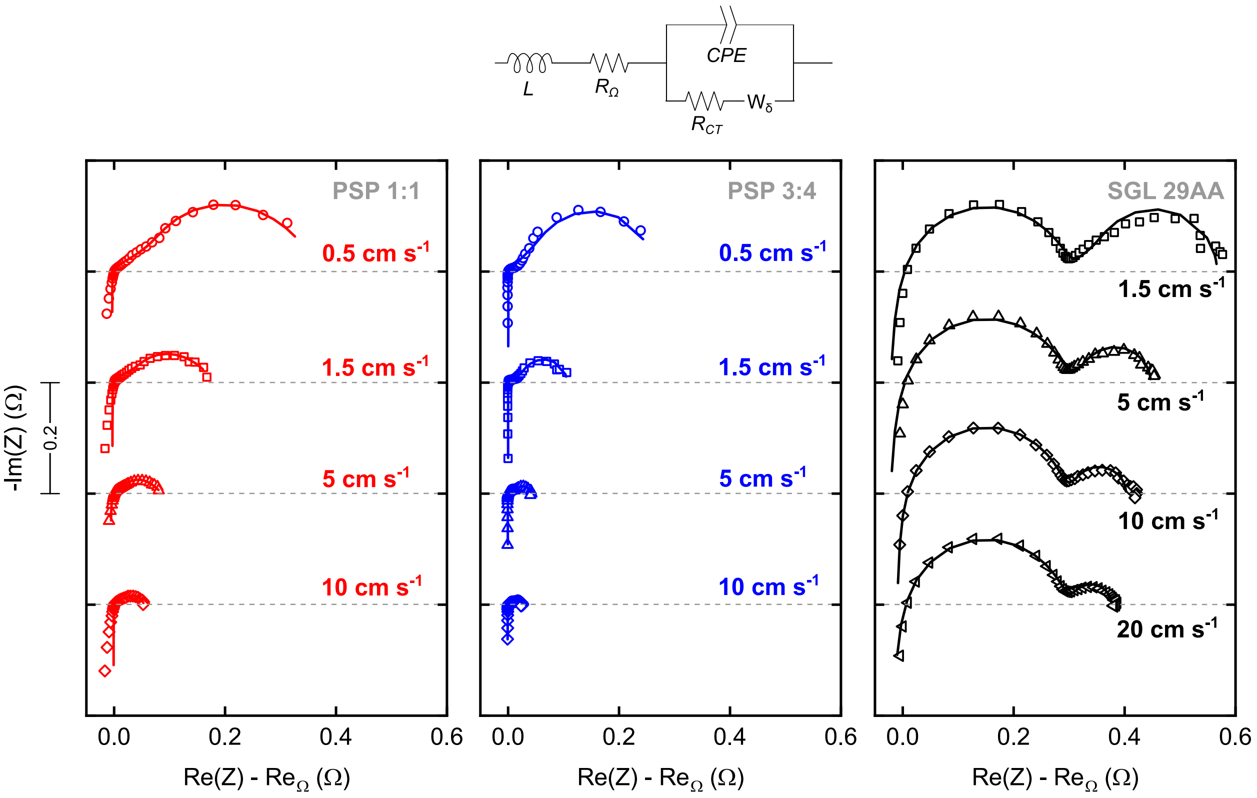

Figure S9. *Representative single-electrolyte electrochemical impedance spectroscopy in iron chloride electrolyte at various linear velocities for PSP-1:1, PSP-3:4, and SGL 29AA electrodes. The equivalent circuit model used to fit the data is shown at the top. The symbols are the collected data, while the lines are from the equivalent-circuit model fits. The 0.5 cm s^{-1} linear velocity was not included for the thinner SGL 29AA due to slow volumetric flow rate leading to variable pump oscillations, rendering EIS spectra uninterpretable.*

Extracted mass transfer resistance from Nyquist plot fittings -

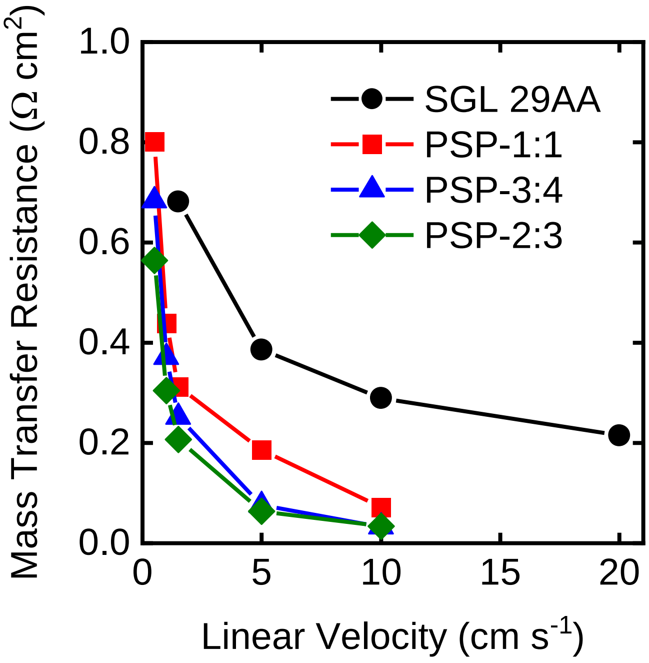

Figure S10. Mass transfer resistance extracted from Nyquist plot fittings as a function of linear velocity.

Capacitance measurements -

The electrochemically active surface area was estimated by charging the double layer in a similar flow cell described in the previous section. To do so, a blank electrolyte was used to avoid the occurrence of any faradaic process. The electrolyte was a 2 M HCl solution, here again a linear electrolyte velocity of 5 cm s^{-1} was used and cyclic voltammetry (CV) was performed between -0.2 V and 0.2 V at 5 different scan rates (20, 50, 100, 150, and 200 mV s^{-1}). The specific capacitance of glassy carbon (23 μF cm^{-2}) was used to estimate the ECSA based on prior art.[10,11]

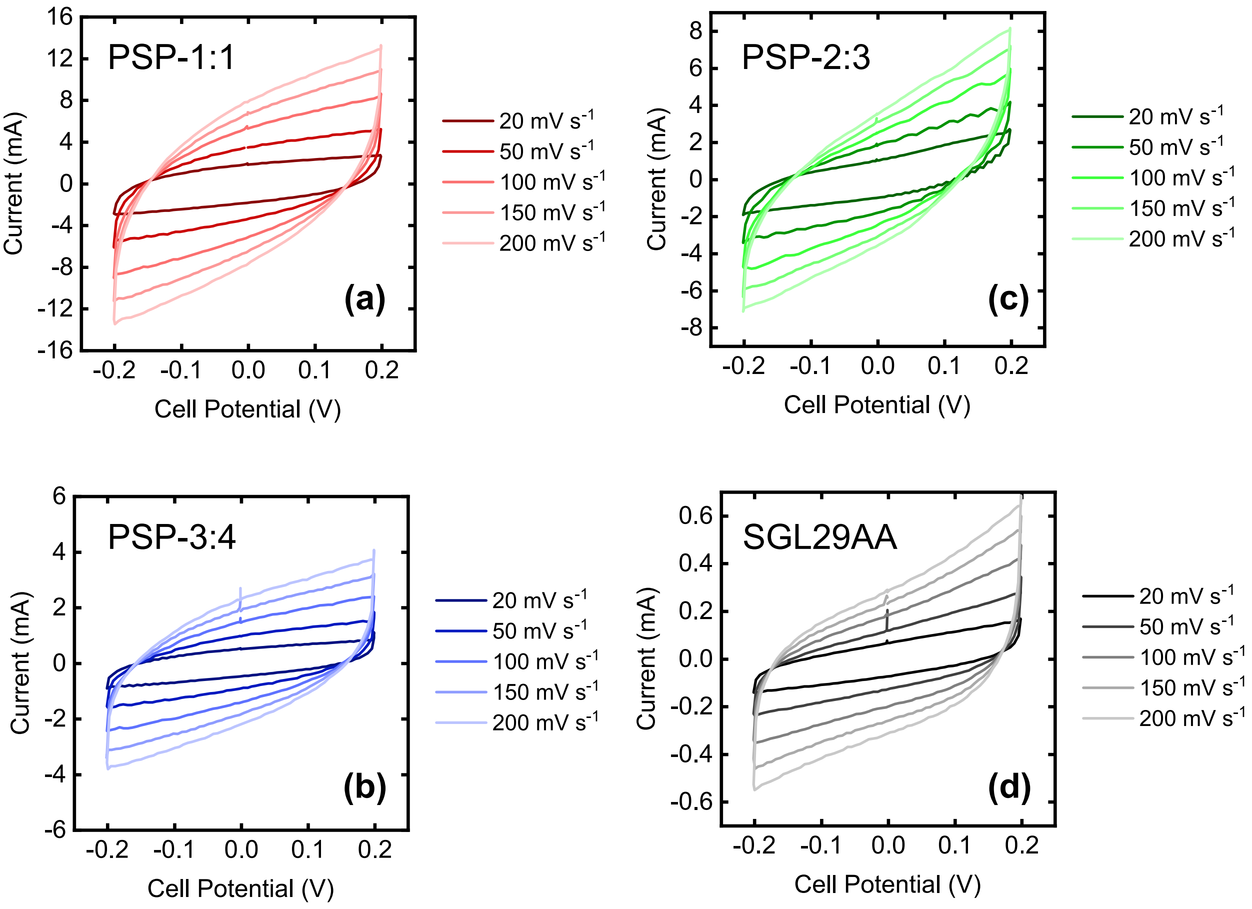

Figure S11. Capacitance measurements taken with 2 M HCl supporting electrolyte at different scan rates for SGL 29AA and the PSP materials.

Pressure drop measurements for interdigitated and flow through flow fields -

Table S6a – *Fitted permeability and Forchheimer coefficient from pressure drop using interdigitated flow fields.*

**Electrode**	**$\kappa \cdot 10^{-11}$ (m^2)**	**$\beta \cdot 10^{-5}$ (m^{-1})**
SGL29AA	32	0.91
PSP-2:3	7.5	0.63
PSP-3:4	3.3	2.0
PSP-1:1	2.2	2.1

Table S6b – *Fitted permeability and Forchheimer coefficient from pressure drop using flow-through flow fields.*

**Electrode**	**$\kappa \cdot 10^{-11}$ (m^2)**	**$\beta \cdot 10^{-5}$ (m^{-1})**
SGL29AA	4.8	64
PSP-2:3	2.0	61
PSP-3:4	1.1	1.7
PSP-1:1	0.76	2.4

Darcy-Forchheimer equation used to fit the pressure drop data:

$$-\frac{dP}{dx} = \frac{\mu}{\kappa} v + \beta \rho v^2$$

Full vanadium redox flow battery cycling experiments –

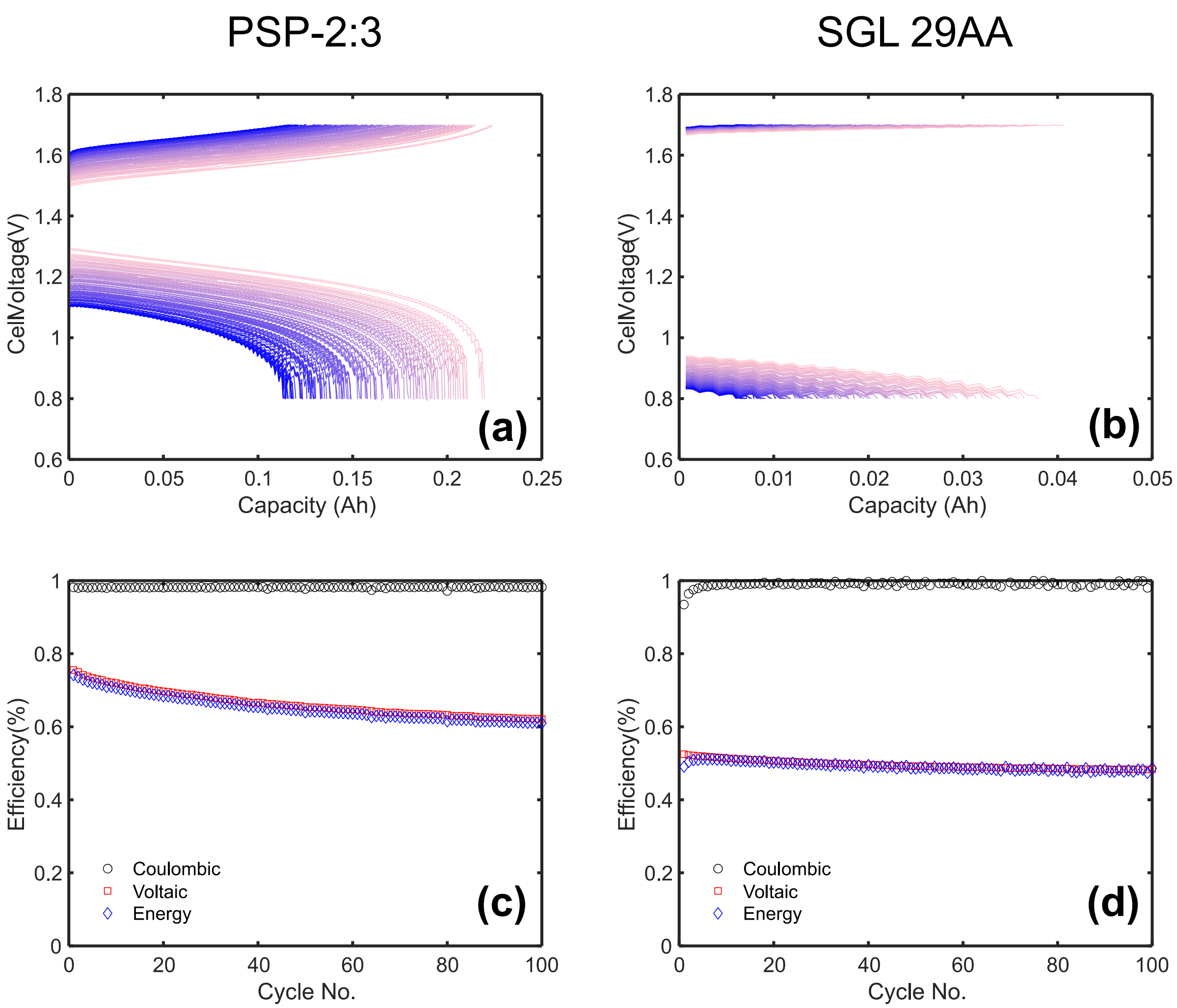

Figure S12. Single vanadium redox flow cell cycling at 100 mA cm^{-2} for PSP-2:3 and SGL29AA. Charge-discharge profiles for the (a) PSP-2:3 and (b) SGL 29AA. Coulombic, voltaic, and energy efficiencies for the (c) PSP-2:3 and (d) SGL29AA.

References

[1] M. S. A. Rahaman, A. F. Ismail, A. Mustafa, *Polym. Degrad. Stab.* **2007**, *92*, 1421.

[2] E. Fitzer, W. Frohs, M. Heine, *Carbon* **1986**, *24*, 387.

[3] S. Liu, M. Kok, Y. Kim, J. L. Barton, F. R. Brushett, J. Gostick, *J. Electrochem. Soc.* **2017**, *164*, A2038.

[4] I. Arganda-Carreras, V. Kaynig, C. Rueden, K. W. Eliceiri, J. Schindelin, A. Cardona, H. Sebastian Seung, *Bioinformatics* **2017**, *33*, 2424.

[5] Z. Gu, M. Z. Bazant, *ArXiv180809804 Cond-Mat Physicsphysics* **2018**.

[6] B. Lesiak, L. Kövér, J. Tóth, J. Zemek, P. Jiricek, A. Kromka, N. Rangam, *Appl. Surf. Sci.* **2018**, *452*, 223.

[7] C. T.-C. Wan, D. López Barreiro, A. Forner-Cuenca, J.-W. Barotta, M. J. Hawker, G. Han, H.-C. Loh, A. Masic, D. L. Kaplan, Y.-M. Chiang, F. R. Brushett, F. J. Martin-Martinez, M. J. Buehler, *ACS Sustain. Chem. Eng.* **2020**, acssuschemeng.0c02427.

[8] I. Bertóti, M. Mohai, K. László, *Carbon* **2015**, *84*, 185.

[9] S. Biniak, G. Szymański, J. Siedlewski, A. Świątkowski, *Carbon* **1997**, *35*, 1799.

[10] K. V. Greco, A. Forner-Cuenca, A. Mularczyk, J. Eller, F. R. Brushett, *ACS Appl. Mater. Interfaces* **2018**, *10*, 44430.

[11] C.-N. Sun, F. M. Delnick, L. Baggetto, G. M. Veith, T. A. Zawodzinski, *J. Power Sources* **2014**, *248*, 560.
